# Can Contrast-Response Functions Indicate Visual Processing Levels?

**DOI:** 10.3390/vision2010014

**Published:** 2018-03-01

**Authors:** Bruno G. Breitmeyer, Srimant P. Tripathy, James M. Brown

**Affiliations:** 1 Department of Psychology, University of Houston, Houston, TX 77204, USA; 2 School of Optometry & Visual Science, University of Bradford, Bradford BD7 1DP, UK; 3 Department of Psychology, University of Georgia, Athens, GA 30602, USA

**Keywords:** contrast response functions, cortical processing level, visual illusion, visual crowding, pedestal masking, lateral masking, feature integration

## Abstract

Many visual effects are believed to be processed at several functional and anatomical levels of cortical processing. Determining if and how the levels contribute differentially to these effects is a leading problem in visual perception and visual neuroscience. We review and analyze a combination of extant psychophysical findings in the context of neurophysiological and brain-imaging results. Specifically using findings relating to visual illusions, crowding, and masking as exemplary cases, we develop a theoretical rationale for showing how relative levels of cortical processing contributing to these effects can already be deduced from the psychophysically determined functions relating respectively the illusory, crowding and masking strengths to the contrast of the illusion inducers, of the flankers producing the crowding, and of the mask. The wider implications of this rationale show how it can help to settle or clarify theoretical and interpretive inconsistencies and how it can further psychophysical, brain-recording and brain-imaging research geared to explore the relative functional and cortical levels at which conscious and unconscious processing of visual information occur. Our approach also allows us to make some specific predictions for future studies, whose results will provide empirical tests of its validity.

## 1. Introduction

Extant psychophysical evidence indicates that cortical processing has a multi-level functional hierarchy [1,2]. At the lowest level, most likely V1 [3], is binocular rivalry produced by interocular competition between, say, orthogonal gratings presented to the two eyes, which is followed by an intermediate level at which metacontrast masking occurs [1]. The metacontrast level is followed by a higher level at which visual crowding occurs, which in turn is followed by the level at which object-substitution masking (OSM) occurs [2] (see [4] for a more complete listing of possible functional levels of stimulus processing). Crowding is believed to rely significantly on neural activity in later cortical levels, an idea reinforced by findings indicating it depends on conscious, perceptrelated levels of processing [5,6,7,8]. Moreover, research on interocular suppression also indicates that during binocular rivalry competitive interactions occur at cortical sites beyond V1 that involve high-level pattern-integrative, in addition to low-level eye-specific, processes [9,10,11]. Some of the psychophysical, and therefore indirect, indicators of functional levels have found their counterpart in a variety of more direct brain-imaging studies of binocular rivalry and metacontrast; with brain-imaging correlates of competitive interactions in binocular rivalry found as early as the lateral geniculate nucleus (LGN) [12,13] and cortical area V1 [3], but also as late as human fusiform facial area (FFA) and parahippocampal place area (PPA) [14]; and metacontrast correlates are most likely found at cortical sites beyond V2 [15,16]. Although brain-imaging studies indicate that the anatomical sites correlating with OSM are found at the post-striate, lateral occipital complex but not at the striate level [17], they have produced mixed results as to what cortical levels contribute to crowding, and how they do so [5,18,19].

To assist the search for levels of processing involved in the various visual phenomena discussed above and additional ones, we suggest on the basis of studies for which data are already available that contrast response functions may prove to be a useful tool. The evidence supporting our rationale for tying processing levels to contrast response functions is outlined below.

## 2. The Relevance of Contrast Response Functions (CRFs)

### 2.1. Interrelatedness of Naturalistic Distributions of Local Contrasts, Cortical CRFs, and the Distinction between Perceptual and Preperceptual Vision

**Figure 1:**
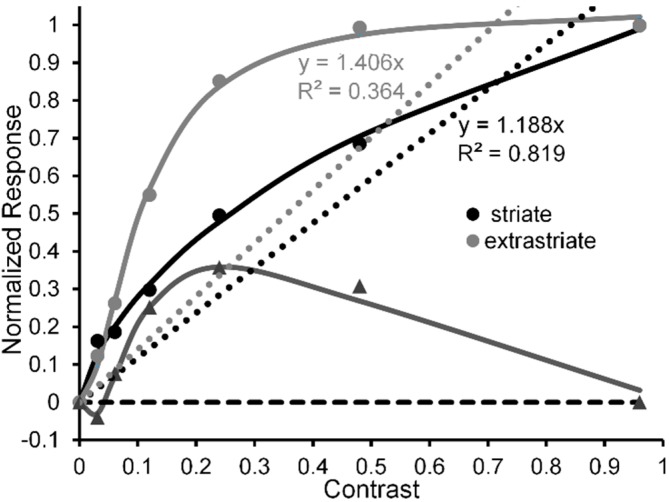
Normalized CRFs, indexed via MEG amplitude, obtained from human striate cortex (black lines and symbols) and extrastriate cortex (grey lines and symbols). Adapted from [24] with permission from the publisher.

Outside of the psychophysical lab human vision is confronted with a multitude of objects and events embedded in ever changing natural scenes. The greater majority of local contrasts in a typical real-world scene fall between 0.0 and 0.3 [20,21,22]. In the striate cortex, the response of neural ensembles tends to track physical contrast by increasing quasi-linearly with stimulus contrast; while at later, extrastriate regions (e.g., see Figure 1) [23], the response of neural ensembles is amplified at low contrasts, rising rapidly over the contrast range of 0 to 0.3 and gradually increases toward maximal (saturated) response thereafter [24,25,26,27,28] (see note [29]). 

In line with, and expanding on, previous interpretations [30], this coincidence of (a) the low values of local contrasts within typical scenes and (b) amplification of the contrast response in mid-and high-level cortical sites for contrasts ≤0.3 (followed by saturation) dovetails nicely with the following observations regarding the distinction between pre-perceptual or pre-conscious and perceptual or conscious vision. (1) At low levels (e.g., V1) in the cortical object-recognition pathway, neural responses are largely stimulus-dependent in that they respond equally to a stimulus even when it is not perceived, whereas at higher levels (e.g., V4 and V5), neural responses become increasingly percept-related, i.e., responding only when the stimulus is perceived [31,32,33]. (2) To maximize extraction of useful perceptual information, the response amplification at low contrasts in the mid- and high-level cortical regions characterizes the transition from low-level linear tracking of an object’s *physical* (luminance) contrast to the registration of brightness contrast as a *perceptual* attribute [34]. These two coincidences raise the following significant question: Can the contrast dependency of visual effects serve to indicate the relative, low/stimulus-dependent vs. high/percept-related, level of processing underlying these effects? i.e., *can CRFs be used to differentiate visual effects due to low-level stimulus-dependent response processes from visual effects due to increasingly high-level percept-related processes?*

### 2.2. Relating CRFs of Individual Neurons to CRFs of Neural Ensembles

One of the problems with assigning quasi-linear responses to the early striate cortex is that *individual neurons* there tend to be characterized quantitatively by CRF functions, most if not all of which are compressively nonlinear in that their responses increase sharply at lower contrasts and saturate at higher stimulus contrasts [35]. Recall, however, that above we referred to *ensembles* of cortical neurons, i.e., a set of neurons at a particular level of cortical processing. No doubt the quasi-linear MEG [24] and fMRI [27] CRFs characterizing striate cortex depend on the combined activity of many hundreds, if not thousands, of individual neurons. So, how does one go from the compressive nonlinear contrast response of individual neurons in striate cortex to a quasi-linear contrast response of neural ensembles in striate cortex?

**Figure 2:**
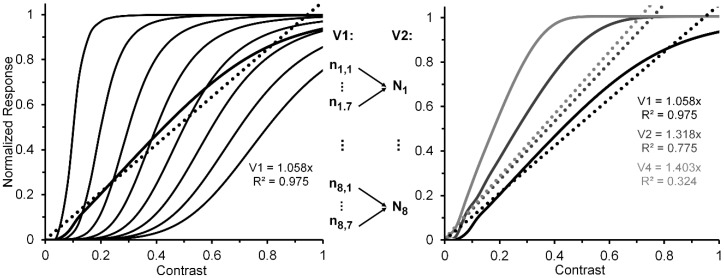
Left panel: Hypothetical normalized CRFs for eight V1 neurons (thin continuous lines), the resulting normalized neuron-ensemble CRF (thick continuous line), and the best linear fit to the neuron-ensemble CRF. See text for details on how the neuron-ensemble CRFs of V2 and V4 were computed. Middle insert: Depiction of how the outputs of seven hypothetical V1 neurons, each with the same CRF, converge on a single V2 neuron. (An analogous process is assumed to describe how seven V2 neurons converge on a single V4 neuron.) Right panel: Hypothetical neuron-ensemble CRFs in V1, V2, and V4.

A reasonable strategy, implicit in Albrecht and Hamilton’s [35] results (see especially their Figure 15) and explicitly suggested by Watson and Solomon [36] (see their Figure 16), is to additively combine the CRFs of several striate neurons whose contrast response functions are characterized by different contrast-gain characteristics. To illustrate this the left panel of Figure 2, via the thin black lines, depicts eight V1 neurons (**n_1_**, …, **n_8_**) whose CRFs shift progressively rightward along the contrast axis. For simplicity, we have characterized their CRFs by using an otherwise identical Naka-Rushton equation [37] (Rmax = 1; α = 5). However, the semi-saturation constant increases, in steps of 0.1, from 0.1 to 0.8. The thick black line in the left panel depicts the normalized CRF obtained by averaging the eight individual CRFs. The hypothetical neural-ensemble function it depicts is not strictly linear; nevertheless, a best fitting linear function, shown by the dotted line, accounts for nearly 98% of its systematic variation. Though simplified, this approach points to a line of reasoning by which more exact modelling can adequately account for an empirically obtained quasi-linear striate CRF, such as that of Hall et al. [24]. 

Let us now turn to the post-striate CRFs. Based on their findings, Tootell et al. [27] argued that extra-striate cortex has a higher contrast sensitivity than striate cortex, which to them suggests that, due to neural response pooling, the contrast sensitivity increases by probability summation in the progressively larger receptive fields of neurons in extrastriate cortex (see middle insert of Figure 2). Within the extrastriate cortex, we can extend Tootell et al.’s approach from one cortical level, say, V2, to the next level, say, V4. To illustrate how this may proceed, the right panel of Figure 2 shows hypothetical normalized CRFs of neural ensembles in V1, V2 and V4. As depicted in the middle insert of Figure 2, the V2 response was constructed by allowing the outputs of seven V1 neurons (**n_i,1_**, …, **n_i__,7_**), each with the same CRF, to converge on one V2 neuron (**N_i_**). Across the eight V1 CRFs, via probability summation this results in the CRF of each of the resulting eight V2 neurons (**N_1_**, ..., **N_8_**) to shift leftward on the contrast axis. The average of these eight V2 CRFs, shown in the dark-grey functions in the right panel, represents the hypothetical V2 neural ensemble response (for details see Appendix A). Now applying the same method to the construction of eight V4 neurons, with V2 neurons providing the seven-fold output converging onto each of the V4 neurons, we arrive at a V4 ensemble response shown by the light-grey functions in the right panel. Because there is successive pooling of responses as one progresses from V1, via intermediate levels V2 and V4, to inferotemporal cortex, we moreover suggest that greater degrees of nonlinearity of the CRF obtained for a contrast-dependent visual phenomenon or effect may be useful indicators that higher levels of processing are involved. Since behavioral data obtained in psychophysical studies using contrast-dependent response indicators could possibly rely on activity of neuronal ensembles at any one, several, or all cortical level of processing, one would be justified in using an obtained CRF that is quasi-linear as indicating the involvement primarily of an early/low level of cortical processing such as V1, with progressively more compressive nonlinear CRFs indicating involvement of progressively higher cortical levels. To illustrate how this may or may not eventuate let us take two flanker paradigms, visual crowding and lateral masking of targets by flanking stimuli, as examples. Relying on normalized magnitudes produced by both paradigms, a significant two-way interaction, particularly at the low to intermediate range of flanker contrast, between flanker paradigm and flanker contrast would indicate that the effects of crowding and lateral masking involve different levels of processing. A lack of interaction would indicate that the two effects involve similar levels.

The rationale of our approach up to now only incorporates the bottom-up feedforward projections from one cortical level (e.g., V1) of visual processing to the next level (e.g., V2). However, besides the feedforward hierarchy from low to high level of cortical processing, a reverse hierarchy giving rise to massive top-down feedback projections from high levels to lower ones [38,39,40,41] also contributes crucially to visual information processing [42,43]. Such back projections from one level to the preceding one, by sharpening and amplifying the responses to the stimulus of neurons at the lower, preceding level [44,45], could thereby also amplify the increasing nonlinearity of the CRFs as one progresses from lower to higher levels in the cortical visual object-recognition pathway (for a possible consequence of such top-down amplification see Section 4.2 below).

That said and having laid out the above groundwork for our approach, we next review three exemplary visual phenomena: illusions, crowding, and masking. Our reason for choosing and limiting our analyses to these phenomena is that for all three (i) several extant studies indicate that they can involve different levels of processing and (ii) at most only a few studies have systematically investigated contrast-dependent effects produced by varying the contrast of the inducers of visual illusions, crowding and masking. Like masking, visual-illusions and crowding have been studied extensively, but mostly without systematic changes of illusion-inducer, flanker contrast, or mask contrast. Since we are especially interested in contrast effects, most of these studies, not directly relevant to the development of our level-specific and contrast-dependent approach, are not included in the following discussions.

## 3. Contrast-Dependent Effects in Visual Illusions, Crowding, and Pedestal Masking

### 3.1. Visual Illusions 

Visual illusions are ubiquitous and have been objects of interest in vision research since at least the ninteenth century. One of them, the tilt illusion, can be obtained when test and inducing gratings are presented concurrently (simultaneous tilt illusion) or as an effect seen in a test grating after adapting to an inducing grating (successive tilt illusion) [46]. Moreover, the tilt illusion can manifest in two ways [47,48,49]: (1) as a repulsive effect in which, for instance, a vertical test grating appears to be tilted slightly in the opposite, clockwise direction when surrounded by an annular grating tilted about 15 deg counterclockwise from vertical; (2) as an assimilative effect in which the vertical test grating appears to be tilted in the same counterclockwise direction as that the surround grating tilted about 75 deg in the counterclockwise direction from vertical. Blakemore, Carpenter and Georgeson [50] proposed that the simultaneous tilt illusion is produced by lateral inhibition among cortical orientation detectors, and in his recent review of the tilt illusion, Clifford [49] has presented a strong case that both the repulsive and assimilative tilt illusions can be explained by a model assuming such inhibition among populations of orientation detectors. On the reasonable assumption that the neurons responding to the orientation of the inducing grating increasingly inhibit the neurons responding to the orientation fo the test graing as the inducing grating’s contgrast increases, one would expect that the repulsive effect of the simultaneous tilt illusion in turn increases as inducing contrast increases. 

**Figure 3:**
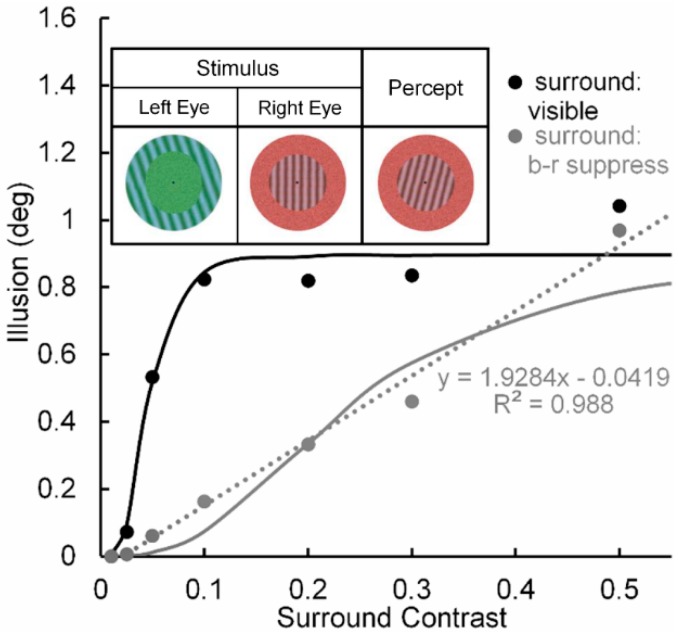
Tilt illusion (in deg) as a function of linearized surround-inducer contrast when the tilted surround is visible (black circles) and when it is b-r suppressed (grey circles). Continuous lines are best fitting Naka-Rushton functions; dotted line is best fitting linear function to the contrast-dependent tilt illusion when the surround is b-r suppressed. Upper inset: Pearson & Gifford’s stimuli presented to observers’ left and right eyes under dichoptic viewing, giving rise to b-r suppression of the left-eye annular grating surround by a right-eye annular noise mask, and the resultant binocularly fused percept. Adapted from [51] with permission from the publisher.

Pearson and Clifford [51] used a circular patch of a vertical test grating surrounded by an annular patch of a tilted inducing grating (see Figure 3, inset) to study the repulsive version of the simultaneous tilt effect. In one condition (Condition 1) they investigated the tilt illusion when the percept of the surround grating presented to the left eye was binocular-rivalry (b-r) suppressed a by noise-mask surrounding a central grating presented to the right eye. In a second, non-rivaling condition (Condition 2) the stimuli in the left and right eye were arranged so that no b-r suppression of the surrounding inducer grating occurred; i.e., it was visible throughout each trial. Pearson and Clifford varied the Michelson contrast of the surround grating from 0.01 to 0.50. The results (see Figure 3) showed that both conditions yielded the tilt illusion, with Condition 1 producing a smaller illusion than Condition 2 across all but the lowest and highest surround contrast. Yet even in Condition 1 the strength of the illusion increased monotonically with inducer contrast, indicating that in this condition the illusion can be generated at a pre-perceptual (pre-conscious) level of processing, somewhere between the level at which binocular rivalry was resolved and the level at which the surround grating registers perceptually in consciousness [52]. 

Significant here is that when the inducing grating is visible, the tilt illusion (see black curve in Figure 3) increases nonlinearly with contrast of the surround grating—steeply in the low contrast range of 0.0 to 0.1 and thereafter saturating. We take this strong nonlinear contrast-amplification trend when the surround grating is visible to indicate, in line with the previous rationale and findings [24,27], that the interaction between the surround grating and the target grating involves mainly high (percept-related levels of processing in the post-striate areas of visual cortex, relative to low (stimulus-dependent) levels of cortical processing in striate cortex. In contrast, when the inducing surround grating is b-r suppressed (see grey curve in Figure 3), the tilt illusion increases in a nearly perfect linear manner, with the best-fit linear function accounting for nearly 99% of systematic variability. Although we take this strong linear trend to indicate, again in line with previous findings and with Pearson and Clifford’s own interpretation, that the interaction between the b-r suppressed inducing grating and the perceived target grating involves mainly a low (pre-perceptual) level of cortical processing in striate cortex, higher-level involvement in the b-r suppression cannot be ruled conclusively.

**Figure 4:**
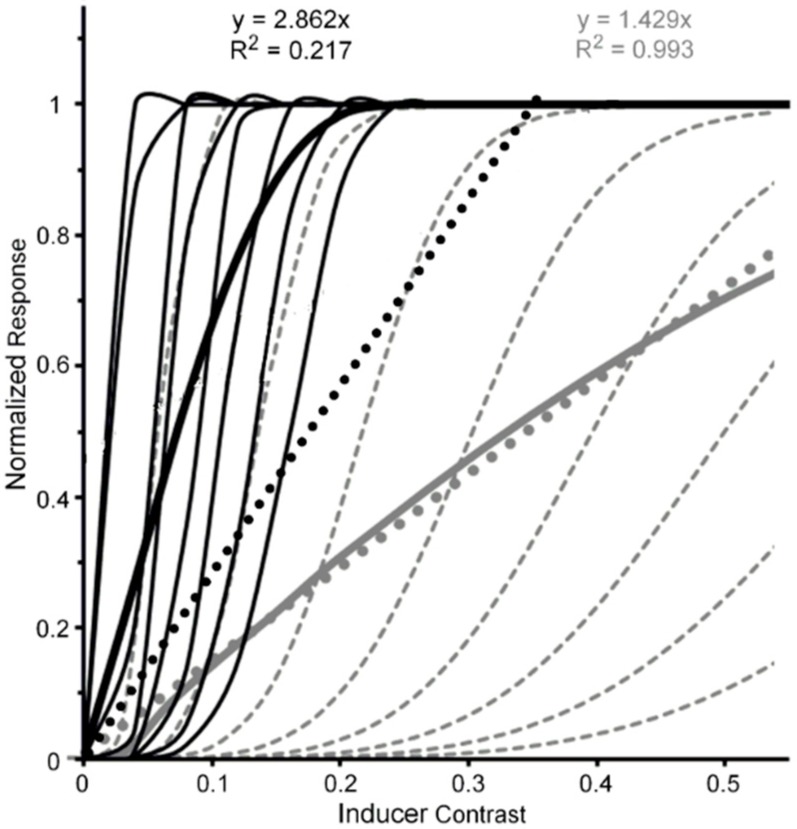
Black lines and symbols: Hypothetical normalized CRFs for eight high-level extrastriate neurons (thin black continuous lines), the resulting normalized neuron-ensemble CRF (thick black continuous line), and the best linear fit (black dotted line) to the neuron-ensemble CRF when tilt-inducing surround is visible. Grey lines and symbols: Hypothetical normalized CRFs for the same eight high-level extrastriate neurons (thin grey dashed lines), the resulting normalized neuron-ensemble CRF (thick grey continuous line), and the best linear fit (grey dotted line) to the neuron-ensemble CRF when tilt-inducing surround is b-r suppressed.

The reason for this tentativeness is the limited range of inducer contrasts (0.0 to 0.5) used by Pearson and Clifford. It is possible that the effect of b-r suppression of the inducer did not cause a change from a higher level to a lower level at which it exerted is effect. One can feasibly argue that b-r suppression caused a reduction of contrast gain at the higher level, resulting in the neural contrast response to shift to the right along the inducer-contrast axis. Figure 4 illustrates how this might happen. The thin black lines depict CRFs of eight high-level neurons when the inducer is visible; while the thick black solid and dotted lines depict the neural-ensemble CRF and its best-fit linear function. Similarly, the thin and dashed grey lines depict CRFs of the same eight high-level neurons when their contrast response is b-r suppressed, with the suppression causing a decrease of contrast gain across all eight neurons. The thick grey solid and dotted lines depict the neural-ensemble CRF and its best-fit linear function. Like Pearson and Clifford’s findings, in this simplified model the high-level neural-ensemble CRF is highly non-linear over the contrast range of 0.0 to 0.55 when the inducer is visible, with the best linear fit accounting for only 57% of its systematic variability across inducer contrast. In comparison, the high-level neural-ensemble CRF is highly linear over the contrast range of 0.0 to 0.55 when the inducer is b-r suppressed, with the linear fit accounting for 99% of its systematic variability across inducer contrast.

Despite this concordance between Pearson and Clifford’s findings and the above model based on high-level b-r suppression, we take this scenario to be unlikely, for the following reasons. (1) Neural correlates of b-r suppression occur as early as striate cortex [3] and even at the precortical LGN level [12,13]. (2) According to Tong and Engel [3], their results indicate that binocular rivalry may be fully resolved at the striate level. (3) As noted by Lee and Blake [10], although high-level pattern rivalry may modulate the low-level, eye-based b-r suppression [3], it is neural processing at the striate levels that is most heavily implicated in the alternating rivalrous phases of suppression and dominance during binocular rivalry [3,53,54]. Hence, on the warranted assumption that the resolution of binocular occurs primarily, if not totally, in V1, it is most likely that when the visibility of the inducer is b-r suppressed, the cortical inducer-target interaction occurs in low-level, linear stimulus-space, whereas when the inducer is perceived, the interaction occurs in high-level, nonlinear percept-space. 

### 3.2. Visual Crowding

**Figure 5:**
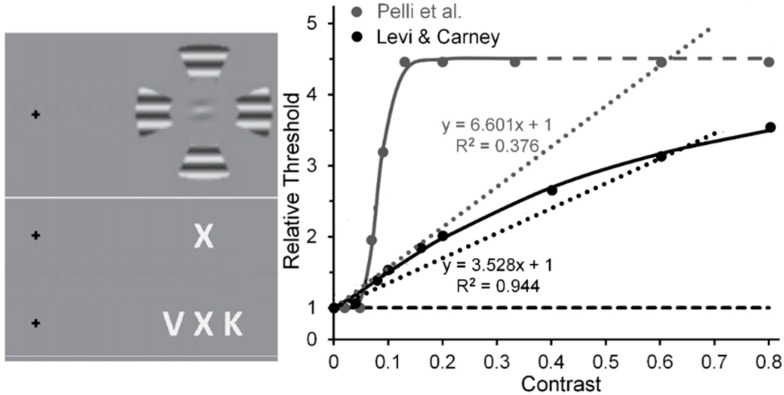
(**Left panel**): (**upper**) Gabor-target and flanker stimuli used by Levi & Carney; (**lower**) letter target and flanker stimuli used by Pelli et al. (**Right panel**): Threshold elevation as a function of flanker contrast. Data points for both studies are accompanied by best fitting Naka-Rushton functions; dotted lines are best fitting linear functions. A dashed line extrapolates the Naka-Rushton function for Pelli et al.’s results beyond their highest Michelson contrast of 0.33. Adapted from [59] and from [60] with permission from the publishers.

Visual crowding, a reduction of visibility of a peripheral target stimulus when it is flanked by nearby distractor stimuli (see Figure 5, left panel), is an important research area per se but also because its effects spill over into many other research areas such as reading, visual search, object and facial recognition, and Gestalt grouping [55,56,57,58]. While, as we noted above, it is recognized that low- and high-level cortical processing can contribute to crowding, it is not clear if and under what conditions the low- and high-level processes contribute differentially to the entire crowding effect. According to our rationale, the following results indicate that these differential contributions can already be assessed, albeit with some caution. 

Levi and Carney [59] used horizontally oriented Gabor patches as targets and wedges of surrounding gratings as flankers in displays akin to that shown in the upper portion of the left panel of Figure 5. Pelli, Palomares and Majaj [60] used letters as targets and flankers in displays akin to that shown in the lower portion of the left panel of Figure 5. To measure the tilt threshold of the target Gabor in the experimental condition of Levi and Carney study, on each trial observers (Os) had to decide, after being presented with two temporal windows—one containing a horizontal Gabor, the other a Gabor tilted slightly from horizontal—which interval contained the more counterclockwise tilted one. The same measure was obtained in a baseline condition where Os viewed the Gabors without the flankers. The flanker’s Michelson contrast was varied from 0.01 to 0.80. Likewise, to provide measures of the contrast threshold of the target letter in Pelli et al.’s study, on each trial Os, guessing when they were not sure, had to identify the flanked target letter by choosing from among several (usually 10) letters that were presented after its 200-ms presentation. Here the flanker’s Michelson contrast [61] was varied from near 0.0 (roughly 0.005) to 0.33. The right panel of Figure 5 shows the results of the two studies. Note that in the Levi and Carney study the threshold elevation for identifying the tilt of the target Gabor increased quasi-linearly as the flanker contrast increases from 0.01 to 0.80 [62], with the linear trend (dotted black line) accounting for 96% of the systematic variability (the Naka-Rushton function (continuous black line) accounted for 99%). What may be less than mere coincidence is the strong agreement between Levi and Carney’s results and, as evident from Figure 1, the quasi-linear increase of normalized MEG amplitudes recorded from human striate cortex by Hall et al. [24]. As noted above, the best linear fit to Levi and Carney’s quasi-linear increase of normalized target thresholds as flanker contrast increased accounted for 94% of the systematic variability; while for Hall et al. the best linear fit to their quasi-linear increase of normalized MEG amplitude accounted for 82% of the systematic variability. In contrast, in Pelli et al.’s study the normalized threshold contrast for identifying the flanked target letter increased clearly nonlinearly (the linear trend (dotted grey line) accounted for only 38% of systematic variability, whereas the nonlinear Naka-Rushton function (continuous grey line) accounts for 99% of systematic variability, with a very steep rise in threshold as flanker contrast increased from 0.04 to 0.16, after which it levels off [63].

### 3.3. Pedestal Masking

**Figure 6:**
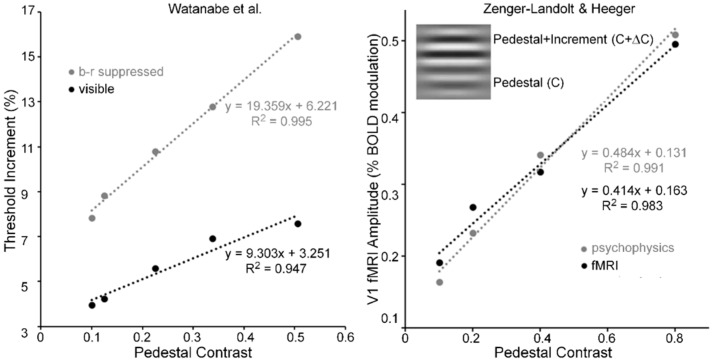
(**Left panel**) threshold increments as a function of pedestal contrast when the pedestal is visible (black circles) and when it is b-r suppressed (grey circles). Dotted lines are best fitting linear functions. (**Right panel**) V1 fMRI amplitude as a function of pedestal contrast when the pedestal is visible. Dotted lines are best fitting linear functions. Inset illustrates a pedestal grating and a pedestal with an increment of its contrast. Adapted from [65] and from [66] with permission from the publishers.

Pedestal masking is a specific case of simultaneous pattern masking, i.e., when target and mask overlap spatiotemporally. As an example of this type of masking, a horizontal Gabor, like that shown in the inset of Figure 6 (right panel), serves as the pedestal (contrast = C) and as the pedestal plus contrast increment (contrast = C + ΔC). For pedestal contrasts of about C = 0.05 and higher, pedestal masking experiments tend to yield linear threshold-increment vs. pedestal-contrast (TvC) functions (e.g., see [64]). Watanabe, Paik and Blake [65] investigated the TvC function when the pedestal was visible and when it was b-r suppressed, and their results are shown in Figure 6 (left panel). Note that while threshold increments when the pedestal was b-r suppressed were about twice as large as those when the pedestal was visible, both functions were linear for pedestal contrasts ranging from 0.1 to 0.5. Relative to the visible pedestal condition, the b-r suppressed pedestal condition thus yielded roughly a two-fold decrease of the sensitivity in the threshold-detecting mechanism, across all pedestal contrasts; this, however, *all the while maintaining the linearity* of the TvC function. 

**Figure 7:**
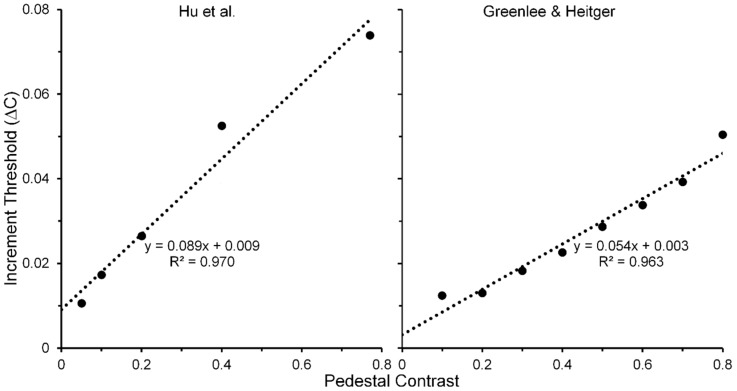
Thresholds for detecting a contrast increment on a pedestal mask as a function of the pedestal contrast. See text for details of how the depicted data points were obtained. See text for details of how the depicted data points were obtained. Adapted from [68] and from [70] with permission from the publisher.

The linearity of Wanatabe et al.’s psychophysical TvC function may be an artifact of their restricted range of pedestal contrasts; hence, their finding alone cannot be taken as the sole evidence for pedestal masking occurring at low, striate levels of processing. Figure 6 (right panel) shows results obtained by Zenger-Landoldt and Heeger [66]. They indicate that a related linearity holds also when psychophysical and fMRI measures of V1 are made during pedestal masking for pedestal contrasts ranging from 0.1 to 0.8 [67]. Moreover, our search of the literature has revealed two other sources of pedestal masking, in which pedestal contrast was varied up to a value of 0.8. The left panel of Figure 7 shows results from Hu, Klein and Carney [68], averaged across two Os and across three spatial frequencies [69] of the pedestal, and the right panel shows results from Greenlee and Heitger [70], again averaged across two Os. Confirming the findings of Zenger-Landolt and Heeger over a similar range of contrasts, both results indicate that the increment threshold for detecting the contrast increment superposed on the pedestal increases nearly linearly with the contrast of the pedestal.

In conjunction with Zenger-Landoldt & Heeger’s findings and those indicating that binocular rivalry is resolved as early as V1 [3,53,54], the fact that linearity of the TvC function was maintained for both visible and b-r suppressed pedestals in the Watanabe et al. study reinforces the conclusion that cortical correlates of pedestal masking are to be found early, and primarily, in V1. Moreover, the combined results of Zenger-Landoldt and Heeger’s, Hu et al.’s, and Greenlee & Heitger’s studies reinforce this interpretation by showing that the linearity of the TvC function can hold for pedestal contrasts ranging up to 0.80. Pedestal contrasts cannot be much higher than 0.90, since one must allow the remaining 0.10 of contrast for the measurement of the threshold increment. A future study in which the pedestal contrast varies from about 0.05 to the maximal allowable value somewhere around 0.90 should resolve any remaining uncertainty as to the linearity of the TvC function. We predict that the TvC function will be strongly linear.

## 4. Discussion, Implications, and Directions for Further Research

### 4.1. A Distinction between Functional and Anatomic Levels of Processing

The rationale of our approach relies primarily on the role of feedfoward hierarchy of cortical visual processing and secondarily on the importance of reverse-hierarchy feedback processing. Much past anatomical research has provided a fairly rich data base supporting cortical feedforward processing and its possible role, but rather sparse studies report on the feedback projections and their possible roles. Nonetheless, rich feedback projections do exist [38,39,40] and their possible roles in visual perception have been noted [41,42,43,44]. However, despite the complexity of cortical visual processing, relative cortical levels of processing have been proposed for several visual paradigms, such as interocular and pattern competition [3,11,14], visual crowding (see Levi [56]) and visual masking [15,16,17]. Even when direct electrophysiological or brain-imaging results are not available to draw conclusions regarding anatomical levels or processing, purely functional levels obtained from psychophysical studies can be distinguished [1,2,4] without, as noted by one of us [4], needing or being able to relate these functional levels to specific anatomical ones. 

### 4.2. Interpretations of Crowding Studies

We have presented evidence that the contrast-response function can be used to assess levels of cortical processing in the simultaneous tilt illusion, visual crowding, and pedestal masking. Regarding first the crowding studies, our analyses and interpretations have the following methodological and theoretical implications. Based on Levi’s extensive review [56] and Pelli et al.’s extensive experimental investigations [60] of visual crowding, the consensus is that crowding is distinct from what is termed ordinary masking, by which is meant masking with spatial overlap of the target and flanker stimuli. However, while, as noted above, crowding is a multi-level phenomenon [71], it is thought to rely primarily on higher, post-striate levels of processing—a possible candidate being V4 [5,56,72,73]—at which integration/conjunction of visual primitives, such as orientation, processed in V1, and perceptual pooling and grouping play an important role [2,18,56,57,73,74]. 

When target and flanking stimuli are similar, crowding typically tends to be stronger [75]. In the case of Levi and Carney’s study, we suggest that, by using the same (2.5 c/deg) spatial frequency and (near-horizontal and horizontal) orientation of the target Gabors and the flanking grating patches, the stimuli favored the component of crowding consisting of lateral inhibitory (masking) interactions between neural units selectively tuned to the same spatial frequencies and orientations. A possible underlying mechanism could be the spatially extensive divisive surround inhibition from outside the classical receptive field of striate neurons [76,77,78]. Particularly relevant here is the suggestion of Polat and co-workers [79,80,81] that lateral masking relies primarily on low-level inhibitory interactions found as early as striate cortex. If, in the Levi and Carney study, such low-level mechanisms were the major contributors to interactions between spatially proximal stimuli, one would expect increases of flanker contrast to produce nearly linear increases of target threshold (see also [82]). On one hand, that would indicate, as noted, that Levi and Carney were investigating a low-level lateral masking component rather than a high-level feature-integrative component of crowding. On the other hand, in Pelli et al.’s study, the clearly nonlinear increase, with strong amplification at low flanker contrasts, implicates the additional contribution of the high-level component to visual crowding. 

**Figure 8:**
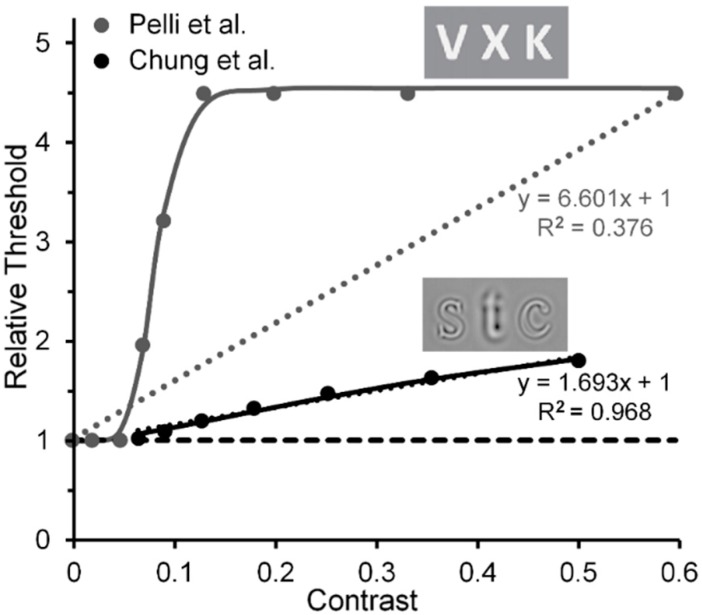
Threshold elevation as a function of flanker contrast. Insets show examples of target and flanker letters used by Pelli et al. and Chung et al. The data points shown for Chung et al. were obtained with spatial-frequency filtered letters having a band pass filter with peak contrast at 1.25 c/letter. Adapted from [60] and from [83] with permission from the publishers.

Another implication is based on comparison of results reported by Chung, Levi and Legge [83] and by Pelli et al. Based on estimates of Chung et al.’s results, it may also be possible to track a transition from the contribution of low-level to high-level visual processes in visual crowding. Chung et al. measured crowding effects using narrow-band spatial-frequency filtered letters, and Pelli et al. measured crowding effects using unfiltered letters (see Figure 8). Note in Figure 8 that with filtered letters the Chung et al. target threshold increases nearly linearly (*R*^2^ = 0.968) with the contrast of the flankers, at least up to a value of 0.5 used by Chung et al. (the Naka-Rushton function yielded a slightly higher *R*^2^ = 0.999) [84]. However, with unfiltered letters it increases clearly nonlinearly in the Pelli et al. study. Aware of the problem raised by Chung et al.’s and Pelli et al.’s restricted flanker-contrast ranges, we nonetheless conjecture that, relative to using unfiltered images such as the letters in Pelli et al.’s study, using filtered letters biases the main contribution to crowding away from high-level processing of letters and toward low-level lateral masking among spatial frequency channels; the narrower the filter bandwidth of the filtered images, the greater the bias towards lower-level processing. 

### 4.3. The Problem of Residual Nonlinearities

Residual nonlinearities in striate cortex [24] that might influence psychophysical tests of low-level vision could arise in two ways. One source of residual nonlinearity could derive from the nonlinear CRFs of magnocellular (M) neurons [85]. It remains to be determined to what extend, if any, this possible source of nonlinearity makes its contribution to experimentally obtained CRFs. A possible hint lies in comparing the V1 fMRI recordings of Zenger-Landoldt and Heeger to those of Gardner et al. [86] and Li et al. [87]. The latter two studies used adapting or pedestal stimuli—checkerboards and sinusoidal grating patches, respectively—which flickered at a rate of 7.5 Hz, whereas the former study used gratings counterphasing at 4 Hz. This difference in flicker rate may have biased the visual system in the latter two studies toward the output of the the more briskly responding M neurons relative to the more sluggish responding parvocellular (P) neurons [88], which would be activated more strongly in the former study. Gardner et al. also obtained increasing adapting effects of flickering checkerboards as their contrast increased from 0.0625 to 0.25, something to be expected since the CRF of M neurons rises steeply and begins to saturate at contrasts of 0.1 to 0.2 [85]. An second source could be cortical feedback. When applying our rationale of systematically varying inducer contrast to visual effects such as crowding or illusions in which on theoretical grounds interactions between the flanker or inducer and the test stimuli rely on low, linear levels of contrast processing (e.g., as in lateral masking and the simultaneous-brightness illusion), one is faced with the effects of residual perceptual nonlinearities. When inducer and target are clearly visible, this is an unavoidable feature of experiments on visual crowding or illusions. One way to minimize the intrusion of such perceptual residues is to use stimuli believed to interact at low levels of visual processing, as in Polat’s lateral masking and in Levi and Carney’s crowding studies. In the Levi and Carney studies, the flankers were clearly visible; and even here small residual nonlinearities of the visual system’s contrast response to the flankers are evident in that the slightly nonlinear Naka-Rushton functions do a slightly better job of accounting for the findings (*R*^2^ = 0.998) than does a best-fit linear function (*R*^2^ = 0.944) (see Figure 5). Since neural activity at high-level, post-striate cortical sites feed back to the striate cortex [89,90], it is possible that such feedback contributes some nonlinearity to the contrast response of striate neurons whenever the inducer is visible. To avoid even this small intrusion of nonlinearity, a more dramatic way to minimize or eliminate the influence of a perceptual residue is to b-r suppress [53] or adaptation suppress [91] the perception of the inducing stimuli, since both types of suppression are thought to prevent processing beyond V1. 

An immediate additional implication is suggested for crowing studies such as Pelli et al.’s that are thought to tap into high-level, percept-dependent cortical processing. As shown in Figure 8, Pelli et al.’s results indicate a strong nonlinear contrast response function of the crowding effect when letters are used as flankers (and, of course, as test stimuli). Adopting the rationale and method of b-r suppression, one would expect that when the flanking letters, as in the Pelli et al. study, are b-r suppressed, the resulting crowding effect will not tap into high-level, percept-related post-striate cortical sites but only into neural processes at the low, striate level. Consequently, we make the strong prediction that here, with the same stimuli used by Pelli et al., the crowding effect should increase gradually and linearly as the flanker contrast increases; whereas it will increase nonlinearly when the flankers are visible.

Related to the crowding phenomenon, above we noted that evidence regarding if and to what extent different anatomical levels of cortical neural networks contribute to crowding points to the involvement of several cortical levels [5,18,19,92]. Our analysis and proposal imply that future brain-recording and brain-imaging studies, in which flanker contrast is systematically varied, may go a significant way toward revealing the factors contributing to the previous mixed findings.

### 4.4. Extensions to Other Visual Phenomena

Another implication is that the proposed approach supports applying our rationale to studying the comparative processing levels of many additional visual phenomena such as visual illusions. We [93] are currently applying it to investigations of the processing levels of simultaneous brightness induction, a version of the tilt illusion different from the one used by Pearson and Clifford, the Ponzo illusion, as well as the White effect [94]. Using our version of the simultaneous tilt illusion, we replicated Pearson and Clifford’s tilt-illusion results obtained when the inducer was visible (see Figure 3). Our results also showed that the magnitude of the tilt illuison increases sharply over inducer contrasts ranging from 0.0 to 0.15 and saturates therafter. According to our proposal, this clear noninearity found in both results indicates that, when the inducer grating is visible, the simultaneous tilt illusion engages lateral inhibitoty interactions among populations of orientation detectors not only at low levels but also at high-levels of visual processing. This would be consistent with the sysematic anatomical maps of neural orientation-specific units found not only in the columnar organization of V1 but also in the interstripe regions of V2 and in V4 [95]. As there are a host of visual effects that reveal important visual processing mechanisms, such as modal and amodal contour completion [89,96], grating induction [97], and phantom gratings [98], to name just a few, one also could readily apply the approach outlined here to these effects. The psychophysical investigations of how their magnitudes change with variation of inducer contrast also could stimulate a search for converging evidence obtained from brain-recording and brain-imaging techniques akin to those used, e.g., by Murray et al. [90] in their study of modal and amodal contours and Zenger-Landoldt and Heeger [66] in their study of pedestal masking. 

One of the leading concerns of visual neuroscience in recent decades has been the distinction between neural correlates of conscious and unconscious vision (NCCVs and NCUVs). Besides visual masking, crowding, and binocular rivalry, there are more than twenty additional noninvasive “blinding” techniques to render stimuli phenomenally invisible or, if visible as in crowding, phenomenally unrecognizable [4,99]. These techniques can be used to establish a functional hierarchy of not only of conscious but also of unconscious visual processing [1,2,4]. The rationale for establishing such hierarchies on unconscious processing can be illustrated by examining the measurable effects of a stimulus varying in contrast but suppressed by different psychophysical blinding methods. For instance, consider an experiment in which three different psychophysical blinding methods are used on the same observers. If a stimulus varying in contrast but rendered phenomenally invisible by low-level b-r suppression yields a linear CRF, one could infer that the invisible stimulus is processed up to the earliest, striate levels of visual cortex but not at later levels. However, if the same stimulus, now rendered invisible by a high-level suppression produced, for example, by OSM [2], yields a strongly nonlinear CRF, one could infer that the invisible stimuli are processed up to a late, poststriate level of visual cortex. In comparison, if backward pattern masking suppresses the visibility of the stimuli and a moderately nonlinear CRF is obtained, one could infer that the invisible stimuli are processed beyond striate cortex level but not up to the late poststriate level. In conjunction with brain-recording and -imaging techniques, such comparative studies could establish how high in the visual cortex unconscious processing of a stimulus (NCUVs) are to be found, and thus rule out processing at that and lower levels as being NCCVs.

Similarly, instead of investigating levels of processing across psychophysical blinding methods, one can investigate them within a given blinding method. For instance, by using contrast-response functions as an indicator one can make predictions about the level of processing in various types of interocular competition. With orthogonal gratings presented interocularly that give rise to eye-seciifc rivary, one would expect to tap into the unconscious processing of spatial-frequency and orientation selective analyzers known to exist as early as the striate level of cortical processing [3,53,54]. Here one would expect to obtain linear CRFs as the contrast of the suppressed stimulus is varied. However, with interocular presentation of faces and houses [14], one would expect to engage the additional competitive interactions between shape/object-specific processing in post-striate FFA and PPA levels of processing. Here, one might expect to obtain nonlinear CRFs when the visibility of one of the contrast-varying stimuli, say, the house, is suppressed by a face of a constant high-contrast. The same rationale readily applies to the many varieties of low- and high-level masking effects [2,100].

Exploring CRFs with psychophysical and brain-recording and -imaging techniques such as EEG, MEG, and optical and fMRI imaging also can reveal the functional levels contributing to the processing for a host of other visual stimuli. Examples are the processing of stimuli defined by equiluminant chromatic contrasts, or equiluminant second-order stimuli, e.g., texture stimuli defined by orientation or size contrast, and dynamic stimuli defined by speed- or direction-of-motion contrasts. 

## 5. Summary and Conclusions

To the question that is the title of our article, we can answer “Yes, they can”. To allow us to make some claims about how differences in contrast-dependent changes of various visual effects might be useful when investigating functional and cortical levels of processing, we have selectively “mined” existing data from only those studies in which inducer contrasts were systematically varied. Based on analyses of these limited studies, we have argued that, for functions relating the magnitude of visual effects to the contrast of stimuli inducing them, differences in the degree of nonlinearity can be useful indicators of corresponding differences in the levels of processing underlying the effects. Of course, since most of these studies have limited ranges of the contrast of the effect-inducing stimuli, our proposal has tentative but nevertheless also suggestive aspects. The important suggestion is that once prior studies are replicated, and novel experiments are devised, each with a generously inclusive range of effect-inducer contrasts, the resulting function relating inducer contrasts to magnitude of visual effects can indicate more conclusively the relative levels of conscious or unconscious processing involved in producing the effects. By defining a broadly applicable research strategy in the field of vision science, our proposal can foster the generation of novel hypotheses guiding further psychophysical, brain-recording, and brain-imaging research.

## Figures and Tables

**Figure A1:**
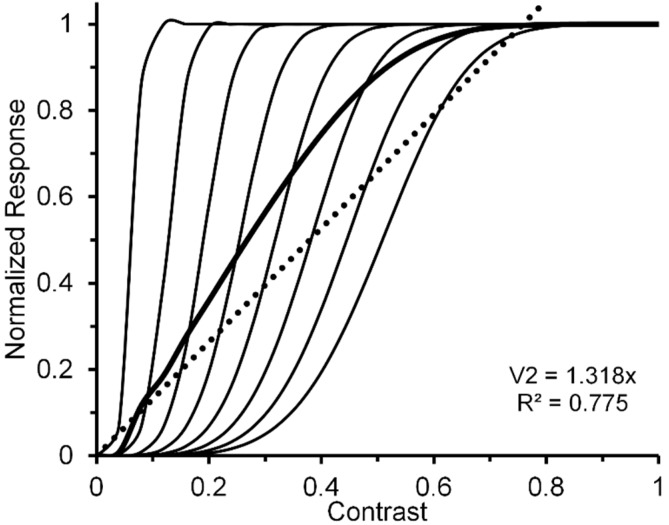
The thin black lines depict CRFs of eight hypothetical V2 neurons each receiving convergent input of seven V1 neurons as shown in the middle panel of Figure 2. Compared to the CRFs of the eight V1 neurons shown in the left panel of Figure 2, the CRFs of the individual V2 neurons shift toward lower contrast values due to probability summation. As is evident from inspection of the thick black line, these steeper increases are transferred by response averaging to the steeper increase of the neuron ensemble of V2 relative to that produced in the neural ensemble of V1 shown in the right panel of Figure 2.

## Appendix A

We develop the rationale for obtaining the contrast-response functions (CRFs) of individual neurons and of an ensemble of neurons. We begin with the assumption that the CRF of a neuron is well approximated by the hyperbolic-ratio Naka-Rushton equation:

(A1) $$R\left(C\right)=R_{\max}\cdot C^{\alpha }/\left(C_{0.5}^{\alpha }+C^{\alpha }\right);$$

where R_max_ is the maximal response magnitude, C is the contrast,${\;C}_{0.5}$ is the contrast at which half of R_max_ is obtained. At which contrast values (Cs) R(C) begins to rise and how rapidly R(C) rises depends jointly on $C_{0.5}$ and the exponent α. The contrast-gain characteristic of the equation is determined by $C_{0.5}^{\alpha }$, with larger values of $C_{0.5}$ or α corresponding to lower contrast gain; the response-gain characteristic of the equation is determined by R_max_, with larger values yielding larger responses. For simplicity, in the following we begin with eight hypothetical V1 neurons; and we assume, for all eight hypothetical neurons: (i) that the response-gain characteristic is the same, with R_max_ = 1; and (ii) that the contrast-gain characteristic, determined by $C_{0.5}^{\alpha }$, has α fixed at a value of 5, but $C_{0.5}$ arying in steps of 0.1 from 0.1 to 0.8 in a hypothetical ensemble of eight V1 neurons.

Since there are eight neurons, we rewrite $C_{0.5}^{\alpha }$, as ${\hat{C}}_{i}^{\alpha }$, *i* = 1, …, 8. Then, by Equation (A1), the CRF for the eight V1 neurons can be given by

(A2) $$R_{V1_{i}}\left(C\right)=R_{\max}\cdot C^{\alpha }/\left({\hat{C}}_{i}^{\alpha }+C^{\alpha }\right);\;i\;=\;1,\;\ldots ,\;8.$$

The resulting graphic representation of the of the corresponding eight V1 neurons’ CRFs is given by the eight thinner continuous lines in the bottom panel of Figure 2. These eight neurons comprise a neural ensemble whose CRF, given by $R_{V1_{\mathrm{ensemble}}}\left(C\right)$, first summed and then normalized by averaging, results in

(A3) $$R_{V1_{\mathrm{ensemble}}}\left(C\right)=\sum_{i=1}^{8}R_{V1_{i}}\left(C\right)/8.$$

The resulting graphic representation of the of the normalized V1 neural-ensemble CRF is given by the thicker continuous line in the left panel of Figure 2. Note that CRF is nearly linear (see thick dotted line: *R*^2^ = 0.975) [101].

We take the outputs of several neurons at one cortical level, e.g., V1, to converge via their (probability) summation [27] on to a single neuron at the next cortical level, e.g., V2, …and iteratively to higher levels. Since the responses of individual V1 are normalized and therefore range from 0 to 1, we can take the contrast-dependent variations of their normalized response magnitudes to be the contrast-dependent variations of their response probabilities. As shown in the middle panel of Figure 2, again for simplicity, we assume that the outputs of seven neurons at one level converge on to a single neuron at the next level. For the convergent probability summation of the outputs of seven V1 neurons on to a single V2 neuron, the CRF for each *i*th V2 neuron is given by:

(A4) $$R_{V2_{i}}\left(C\right)=1-{\left(1-R_{V1_{i}}\left(C\right)\right)}^{7};\;i\;=\;1,\;\ldots ,\;8.$$

In Figure A1, the CRFs of the resulting eight individual V2 neurons are depicted by the thinner continuous lines. These eight V2 neurons comprise a neural ensemble whose CRF, given by $R_{V2_{\mathrm{ensemble}}}\left(C\right)$, again first summed and then normalized by averaging, resulting in

(A5) $$R_{V2_{\mathrm{ensemble}}}\left(C\right)=\sum_{i=1}^{8}R_{V2_{i}}\left(C\right)/8.$$

The corresponding graphic representation of the of the normalized V2 neural-ensemble CRF is given by the thicker continuous line in the upper panel of Figure A1. Since, compared to the individual neural V1 CRFs, the individual V2 neural CRFs shift toward lower contrast values (increased contrast gain), the V2 neural-ensemble CRF rises more quickly at low contrasts than the V1 counterpart and therefore also is more nonlinear.

By iteration we can obtain similar individual and ensemble neural responses, via seven V2 neurons converging on a single V4 neuron. The resulting V1, V2, and V4 normalized neural ensemble responses along with their best linear fit are shown in the right panel of Figure 2. The degree of nonlinearity, indicated by the progressive reductions of *R*^2^ values yielded by the best linear fits (0.975, 0.775, and 0.324), increases as one progresses from cortical level V1 to level V4.
